# Supplementary material for: Model-Based Assessment of the Role of Uneven Partitioning of Molecular Content on Heterogeneity and Regulation of Differentiation in CD8 T-Cell Immune Responses
Source: Front Immunol. 2019 Feb 19;10:230. doi: 10.3389/fimmu.2019.00230 (PMC6392104; doi:10.3389/fimmu.2019.00230)
Supplement: Supplementary File 1 — Parameter value tables and sensitivity analysis to parameter Eomesth. [file Data_Sheet_1.PDF]

Supplementary Material: Model-based assessment of the role of uneven partitioning of molecular content on heterogeneity and regulation of differentiation in CD8 T-cell immune responses

1 PARAMETER VALUES

Parameter	Description	Value	Reference
A_T	target area of CD8 T-cell	144 μm^2	Gao et al. (2016)
A_{APC}	target area of APC	2250 μm^2	calibration
P_T	target perimeter of CD8 T-cell	48 μm	calibration
λ_{pm}	weight of perimeter constraint	10	calibration
λ_{area}	weight of area constraint	10	Prokopiou et al. (2014)
T	temperature	10	Prokopiou et al. (2014)
$J_{APC,m}$	APC-medium contact energy	30	Prokopiou et al. (2014)
$J_{T,m}$	CD8 T-cell-medium contact energy	30	Prokopiou et al. (2014)
$J_{APC,APC}$	APC-APC contact energy	100	Prokopiou et al. (2014)
$J_{APC,NPA}$	APC-naive or preactivated CD8 T-cell contact energy	35	Prokopiou et al. (2014)
$J_{APC,AEM}$	APC-activated, effector or memory CD8 T-cell contact energy	100	calibration
$J_{T,T}$	CD8 T-cell-CD8 T-cell contact energy	100	Prokopiou et al. (2014)
$v(\sigma_{APC})$	weight of motility energy for an APC	25	Gao et al. (2016)
$v(\sigma_{PA})$	weight of motility energy for a preactivated CD8 T-cell	0	Gao et al. (2016)
$v(\sigma_T)$	weight of motility energy for a CD8 T-cell (except preactivated)	250	Gao et al. (2016)

Table S1: Estimated parameter values for the Cellular Potts model. Weights, temperature and energies are dimensionless parameters.

Parameter	Value	Unit	Reference
Strengths of feedbacks			
λ_{R1}	0.58	mol L^{-1} min^{-1}	calibration
λ_{R2}	0.007	min^{-1}	calibration
λ_{E1}	0.001	min^{-1}	calibration
λ_{T1}	0.07	mol L^{-1} min^{-1}	calibration
λ_{T2}	0.06175	mol L^{-1} min^{-1}	calibration
λ_{T3}	35	mol L^{-1}	calibration
λ_{c1}	0.096	mol L^{-1} min^{-1}	calibration
λ_{c2}	0.07	L mol^{-1}	calibration
λ_{c3}	0.23	/	calibration
λ_{c4}	1.4	min^{-1}	calibration
λ_{E2}	0.073	L mol^{-1}	calibration
λ_{E3}	0.06	mol L^{-1} min^{-1}	calibration
λ_{E4}	0.09	mol L^{-1} min^{-1}	calibration
λ_{E5}	20	/	calibration
λ_{E6}	10	mol L^{-1}	calibration
λ_{E7}	0.035	L mol^{-1}	calibration
Degradation rates			
k_R	0.0077	min^{-1}	calibration
k_e	0.0154	min^{-1}	calibration
k_T	0.00051	min^{-1}	calibration
k_F	0.003	min^{-1}	calibration
k_c	0.0038	min^{-1}	Prokopiou et al. (2014)
k_E	0.0035	min^{-1}	calibration
Association/dissociation rates			calibration
μ^{+}_{IL2}	6×10^{6}	L mol^{-1} min^{-1}	calibration
μ^{-}_{IL2}	0.12	min^{-1}	calibration
μ^{+}_{F}	0.0002	L mol^{-1} min^{-1}	Gao et al. (2016)
μ^{-}_{F}	0.004	min^{-1}	Prokopiou et al. (2014)
k_E	0.0035	min^{-1}	calibration
Other			
λ_F	4.2×10^{-5}	mol L^{-1} min^{-1}	Prokopiou et al. (2014)
n	3	/	calibration

Table S2: Parameter values for System (1)-(6).

Parameter	Value	Unit	Reference
λ_{R3}	5×10^{-9}	mol L^{-1} min^{-1}	calibration
λ_{R4}	15	mol L^{-1}	calibration
λ_{T4}	0.08	L mol^{-1}	calibration
λ_1	3×10^{-8}	L mol^{-1}	calibration
D	1.776	μm^2 min^{-1}	calibration
δ	0.187	min^{-1}	Gao et al. (2016)

Table S3: Parameters values for equation (7).

Thresholds	Value	Unit
$IL2R_{th}$	62.9	mol L^{-1}
$Tbet_{th}$	16.8	mol L^{-1}
$Eomes_{th}$	16 (15.6 in Section 3.4)	mol L^{-1}
$Caspases_{th}$	19.42	mol L^{-1}

Table S4: Threshold values for differentiation and death of CD8 T-cells.

2 SENSITIVITY ANALYSIS TO PARAMETER $EOMES_{TH}$

In this paper, we enriched previous models (Gao et al., 2016; Prokopiou et al., 2014) in order to allow differentiation into memory cell. In our model, this differentiation occurs when the concentration of protein Eomes in an activated or effector cell crosses the threshold $Eomes_{th}$ (see Section 2.3.1). Here, we discuss how sensitive our model is to this parameter. Sensitivity to other parameters has been investigated in Gao et al. (2016) and Prokopiou et al. (2014).

It is no surprise that the number of memory cells at the end of the response (D25 p.i) is highly sensitive to $Eomes_{th}$ value, as shown on Figure S1.A. Indeed, for low values of $Eomes_{th}$, the memory precursor cells identified in Section 3.3 differentiate early while for higher values of $Eomes_{th}$ they can accomplish a few additional rounds of division before their Eomes concentrations are sufficiently high to trigger memory differentiation, leading to bigger memory cell populations. Indeed, Figure S1.B illustrates that the lower the value of $Eomes_{th}$, the sooner the pool of memory CD8 T-cells stops to expand.

We can see on Figure S1.C that the number of cells at the peak of the response (i.e. the maximal expansion size reached by the CD8 T-cell population) is less sensitive to the value $Eomes_{th}$. However, it appears that the peak population size slightly increases with the value of $Eomes_{th}$. This is mainly due to the reason exposed above, i.e. memory precursor effector cells proliferate more before their differentiation into non-dividing memory cells.

It is also worth noting that if $Eomes_{th}$ is too low, CD8 T-cells can differentiate into memory cells even if their molecular content does not match with the expected properties of memory cells (survival and low cytotoxicity). Indeed, if $Eomes_{th}$ is 90% (resp. 95%) of its base value (see Table S4), an average of 3.6 (resp. 0.4) memory cells die during the response (mean over 5 simulations, results not shown) while for the base value of $Eomes_{th}$ or upper values there is no memory cell death.

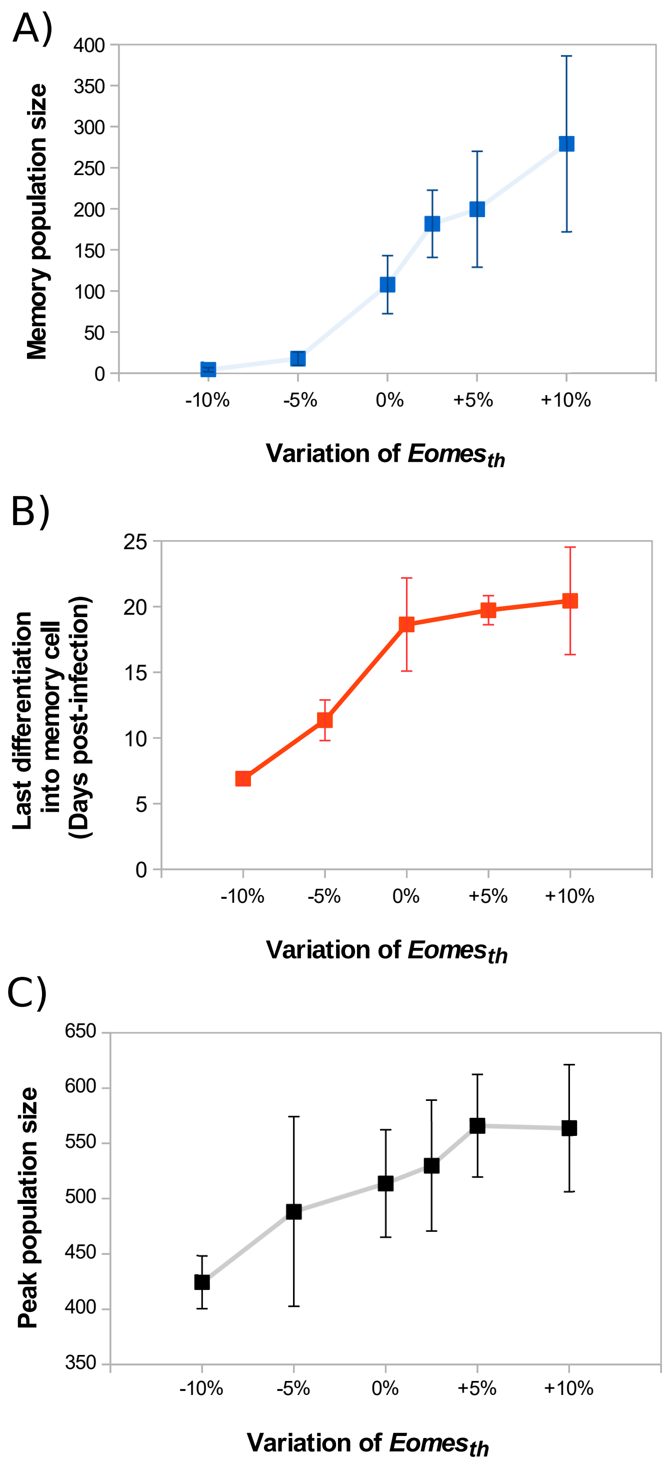

Figure S1: Sensitivity analysis to parameter $Eomes_{th}$. (A) Number of memory cells at the end of the response D25 p.i., (B) day at which the last differentiation into memory cell occurs and (C) number of CD8 T-cells at the peak of the response as functions of the parameter $Eomes_{th}$ (mean +/- standard deviation over 5 simulations). Results are shown for variations of -10%, -5%, $+2.5\%$, $+5\%$ and $+10\%$ from the base value of $Eomes_{th}$ (Table S4).

Figure S2 represents the mean concentration of IL2 receptors, Tbet and Eomes among the CD8 T-cell population from D4 to D15p.i., for different values of $Eomes_{th}$. Since Eomes expression is associated with memory phenotype, it is no surprise that Eomes concentration increases more slowly, or even decreases, for low values of $Eomes_{th}$. On the opposite, high values of $Eomes_{th}$, associated with big memory cell populations, lead to decreasing Tbet concentrations.

REFERENCES

Gao X, Arpin C, Marvel J, Prokopiou SA, Gandrillon O, Crauste F. IL-2 sensitivity and exogenous IL-2 concentration gradient tune the productive contact duration of CD8+ T cell-APC: a multiscale modeling study. *BMC. Syst. Biol.* **10** (2016) 77. doi:10.1186/s12918-016-0323-y.

Prokopiou SA, Barbarroux L, Bernard S, Mafille J, Leverrier Y, Arpin C, et al. Multiscale modeling of the early CD8 T-cell immune response in lymph nodes: An integrative study. *Computation* **2** (2014) 159–181. doi:10.3390/computation2040159.

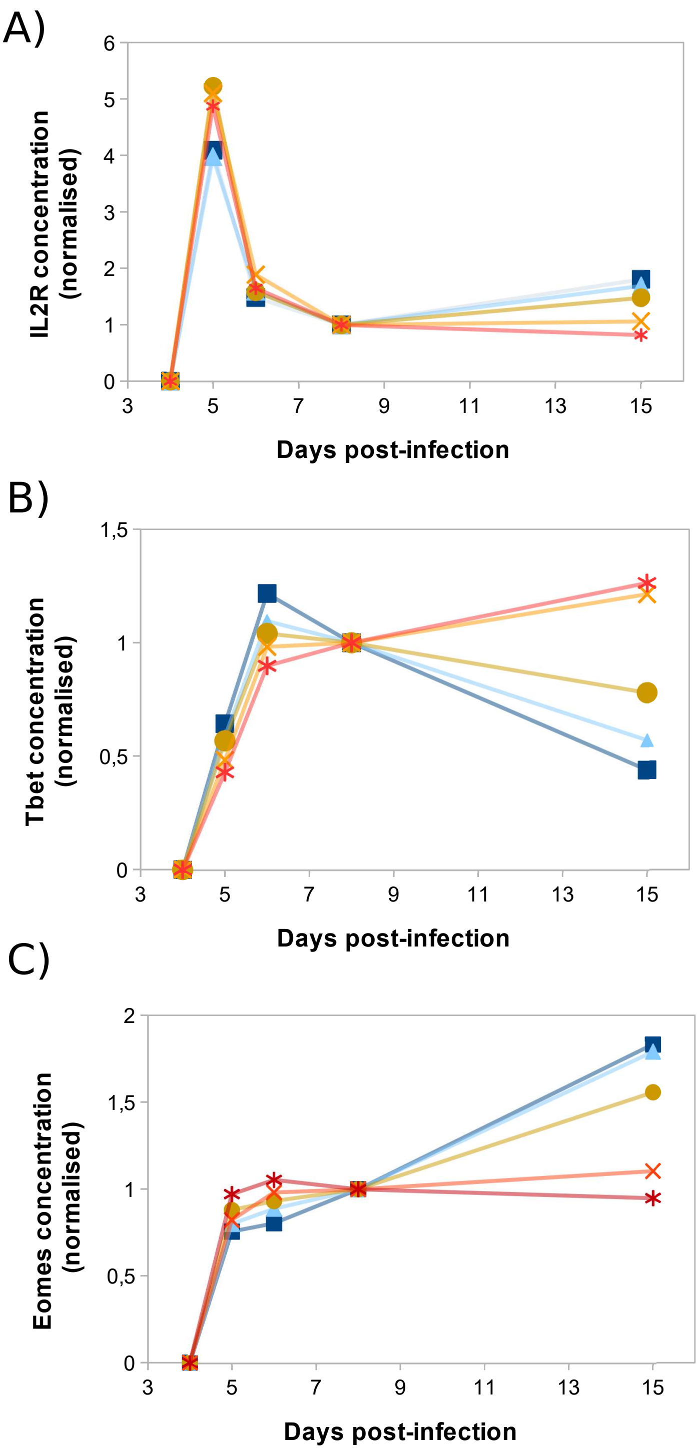

Figure S2: Mean concentration of (A) IL2 receptors, (B) Tbet and (C) Eomes among the CD8 T-cell population normalised by the concentration value D8 p.i. for different values of $Eomes_{th}$ (mean over 5 simulations). Results are shown for variations of -10% (red stars), -5% (orange crosses), 0% (yellow discs), $+5\%$ (light blue triangles) and $+10\%$ (dark blue squares) from the base value of $Eomes_{th}$ (Table S4).
